# Supplementary material for: Prevention of Blood Incompatibility Related Hemagglutination: Blocking of Antigen A on Red Blood Cells Using In Silico Designed Recombinant Anti-A scFv
Source: Antibodies (Basel). 2024 Aug 1;13(3):64. doi: 10.3390/antib13030064 (PMC11348219; doi:10.3390/antib13030064)
Supplement: Supplementary file 1 [file antibodies-13-00064-s001.zip › antibodies-3092670-supplementary.pdf]

Prevention of blood incompatibility related hemagglutination: Blocking of antigen A on red blood cells using *in silico* designed recombinant Anti-A scFv

Saturation of antigen-A and hemolysis of RBCs

A preliminary test was conducted to determine the optimal concentration of RBCs covering the range of dilutions starting from 1 nM to 500 uM. For this cells/ul of RBCs were determined and different volumes of RBCs (0.5, 1, 5, and 10 µL) were taken. It was assumed that the number of cells in each volume were a direct multiple of volume (ul) (table 1). Two experiments were performed with each volume of RBCs using highest dilution 1 nM and lowest dilution 500 uM.

1. **Incubation with Highest Dilution (1 nM):** Each RBC volume was resuspended in 1 nM anti-A scFv solution and incubated for 1 hour at 37 °C with shaking. After incubation, an indirect ELISA was performed to quantify scFvs attached to the RBCs.
2. **Incubation with Lowest Dilution (500 µM):** Each RBC volume was resuspended in 500 µM anti-A scFv solution and incubated for 1 hour at 37 °C with shaking. The supernatant was collected for indirect ELISA analysis, and the RBCs were assessed for hemagglutination.

Table S1: Number of cells of RBCs in each volume

A+	
**Volume (ul)**	**Cells/ul**
0.5	2.5×10^6
1	5.1×10^6
5	$5.1 \times 10^6 \times 5$
10	$5.1 \times 10^6 \times 10$

Table S2: Absorbance in OD of attached scFvs on RBCs measured through microplate reader

A+	
**Volume (ul)**	**OD at 450 nm**
0.5	0.0*
1	0.008
5	0.150
10	0.289

*Concentrations not in detectable range

Table S3: Percentage of scFvs left in supernatant of lowest dilution (500 uM) and hemagglutination of RBCs after incubation.

A+			
**Volume (ul)**	**Percentage %**		**Hemagglutination**
	**Initial**	**Final**	
0.5	100	44	No
1	100	29	No
5	100	21	Yes
10	100	13	Yes

The lowest volume of RBCs in which attached scFvs were detectable when incubated in 1 nM dilution (table 2) and the highest volume of RBCs, in which no hemagglutination was observed when incubated in 500 μM dilution, was selected for subsequent experiments (table 3).
